# Human Case of Leptospirosis During a Canine Disease Outbreak — Wyoming, 2023

**DOI:** 10.15585/mmwr.mm7327a1

**Published:** 2024-07-11

**Authors:** Brittney Waranius, Courtney Tillman, Clay Van Houten, Alexia Harrist, Rose Digianantonio, Hallie Hasel, Christine Atherstone, Emily Curren

**Affiliations:** ^1^Epidemic Intelligence Service, CDC; ^2^Wyoming Department of Health; ^3^Wyoming Livestock Board, Cheyenne, Wyoming; ^4^Division of High-Consequence Pathogens and Pathology, National Center for Emerging and Zoonotic Infectious Diseases, CDC.

**Summary**

**What is already known about this topic?**

Leptospirosis, a zoonotic bacterial disease that results from contact with body fluids of infected animals, occurs worldwide and can lead to severe illness in humans and animals.

**What is added by this report?**

A case of human leptospirosis was reported in Wyoming, a historically low-incidence state with a cold and semiarid climate considered to lower the probability for disease emergence. Coordination by human- and animal-focused public health agencies facilitated epidemiologic linkage of the case to a canine outbreak through occupational exposure.

**What are the implications for public health practice?**

Leptospirosis should be considered in the differential diagnosis of persons with occupational exposure to animals and clinically compatible signs and symptoms, including fever, chills, myalgia, nausea, vomiting, diarrhea, headache, calf pain, and conjunctival suffusion, irrespective of geographic location.

## Abstract

Leptospirosis is a zoonotic bacterial disease spread through the urine of infected animals; the typical incubation period is 5–14 days. In approximately 90% of human cases, illness is asymptomatic or mild, characterized by fever, chills, myalgia, nausea, vomiting, diarrhea, headache, calf pain, and conjunctival suffusion, but severe illness can progress to multiorgan dysfunction and death. Although Wyoming is considered a low-risk area for leptospirosis because of its cold and semiarid climate, the Wyoming Department of Health was notified of a probable human case in August 2023, the first reported in the state since 1983. The patient had occupational exposure to dogs but did not report other risk factors. The same week that the human patient’s illness began, public health authorities received notification of an increase in canine leptospirosis cases. Public health authorities investigated to determine potential sources of infection, identify additional cases, and recommend control measures. After public health outreach activities were implemented, canine vaccination practices changed substantially in the affected city: a survey conducted after the outbreak revealed that all responding veterinary clinics in the affected city were recommending the vaccine more frequently to dog owners and reporting higher levels of owner compliance with vaccination recommendations. Increased vaccination coverage offers protection from leptospirosis for both dogs and persons exposed to them. Leptospirosis should be considered in the differential diagnosis of persons with occupational exposure to animals and clinically compatible signs and symptoms, including fever, chills, myalgia, nausea, vomiting, diarrhea, headache, calf pain, and conjunctival suffusion, irrespective of geographic location.

## Introduction

Leptospirosis is an acute zoonotic bacterial illness characterized by fever, chills, myalgia, nausea, vomiting, diarrhea, headache, calf pain, and conjunctival suffusion (i.e., redness without inflammatory exudates). The incubation period is normally 5–14 days, and 90% of cases in humans are asymptomatic or result in mild, self-limited illness (*1*). However, severe illness can progress to multiorgan dysfunction and death. Factors associated with severe disease include high levels of leptospiremia, delayed antimicrobial treatment, infection with *Leptospira interrogans* serogroup *Icterohaemorrhagiae*, chronic hypertension, chronic alcoholism, and age ≥60 years (*2*–*4*). Oral antibiotics are the treatment of choice for mild illness; patients with severe cases might require hospitalization with intravenous antibiotics and aggressive supportive care (*1*). In August 2023, the Wyoming Department of Health (WDH) was notified of a human case of leptospirosis, the first case reported in the state since 1983 (*5*). Leptospirosis is more common in wet and warm regions, and Wyoming is typically considered a low-risk location because of its dry and semiarid climate (*6*).

## Investigation and Results

### Data Source

WDH staff members reviewed medical records, interviewed the patient, and reviewed reportable conditions data* to monitor for additional cases. The office of the State Animal Health Official (SAHO) obtained histories for canine leptospirosis cases, reviewed veterinary records, and interviewed veterinary clinic staff members. This activity was reviewed by CDC, deemed not research, and was conducted consistent with applicable federal law and CDC policy.^†^

### Case Report

The patient reported initial signs and symptoms of body aches, fever, nausea, and sweating. Two days later, after briefly losing consciousness, the patient was treated in a hospital emergency department with atropine and intravenous fluids for vasovagal syncope and released. The illness worsened during the subsequent 2 days, and included symptoms of calf pain, shortness of breath, cough, headache, conjunctival hyperemia, lower extremity edema, lightheadedness, and reported “brain fog.” The patient reported no exposure to common risk factors, including standing water or mud, or participation in activities such as traveling, hunting, and adventure sports. The only reported risk factor was occupational exposure to dogs.

After the first hospitalization, the patient learned about several canine leptospirosis cases from a colleague. The patient sought follow-up care with a primary health care provider on day 5 of illness and was hospitalized again on day 6 with signs of pleural effusion, hypoxemia, and acute kidney injury. The patient did not have a known connection to a canine case but was occupationally exposed to body fluids from multiple dogs, including three that died from unknown causes. Despite experiencing illness consistent with leptospirosis and communicating occupational risk to multiple health care providers, the patient did not receive testing until day 8 of illness, at which time immunoglobulin M antibodies to *Leptospira* sp*.* were detected.^§^ The patient began treatment with oral doxycycline (100 mg twice daily for 7 days) on day 11 of illness and improved sufficiently to be released 1 day later.

### Reports of Increase in Canine Leptospirosis Cases

The day of the patient’s illness onset, a local veterinary clinic diagnosed leptospirosis in three dogs. Canine leptospirosis is rarely diagnosed in Wyoming and is not a reportable disease in the state. However, the veterinarian was concerned that the cases represented an increase in morbidity and therefore, reported the cases to SAHO.

SAHO requested that veterinary clinics statewide voluntarily report canine leptospirosis cases, and during August–October 2023, a total of 13 canine cases were reported. Veterinary records and interviews with veterinary clinic staff members indicated that the ill dogs had nonspecific signs and symptoms, including vomiting, lethargy, and decreased appetite. All the ill dogs experienced azotemia (increased levels of blood urea nitrogen and serum creatinine, resulting from kidney injury) and four were euthanized or died of severe disease. Three dogs met confirmed leptospirosis case criteria and 10 met probable case criteria^¶^ (*6*). Because none of the deceased dogs associated with the patient had been tested for leptospirosis, they were not among the dogs with confirmed or probable cases.

Veterinary records indicated that canine cases were geographically dispersed throughout the city where the patient worked. Five affected dogs were epidemiologically linked to the same boarding kennel during August–September. One additional dog was potentially exposed through ingestion of standing water. Seven dogs could not be linked to high-risk activities or locations. However, wet conditions might contribute to increased environmental persistence of *Leptospira*, and the affected region experienced nearly double its average precipitation during the 3 months preceding the outbreak (*6*,*7*), suggesting that dogs might have been exposed in the general environment.

Three ill dogs received microagglutination testing results demonstrating high antibody titers (≥1:800) to vaccine-preventable serovars. However, none of the dogs affected during this outbreak was up to date on leptospirosis vaccination.

## Public Health Response

### Case Surveillance

WDH conducted interviews with close contacts and work colleagues of the patient and SAHO conducted interviews with staff members at facilities visited by infected dogs. No additional human cases were reported or identified through interviews.

### Control Activities

WDH alerted local health care providers about the outbreak to encourage prompt testing of persons with clinically compatible signs and symptoms, such as fever, chills, myalgia, nausea, vomiting, diarrhea, headache, calf pain, or conjunctival suffusion. SAHO notified veterinary clinics and boarding facilities. WDH and SAHO also inspected the epidemiologically linked boarding kennel to evaluate vaccination policies, quarantine procedures, and cleaning and disinfection protocols. Although the facility required vaccination of boarded dogs against multiple diseases, it did not require vaccination against leptospirosis. Recommendations for preventing disease transmission were provided, including requiring leptospirosis vaccination for all dogs, eliminating standing water, following appropriate cleaning and disinfection protocols, isolating ill dogs, and using best practices for personal protective equipment.**

### Public Outreach

WDH published a news release^††^ and conducted interviews with media outlets to notify the public of the outbreak. SAHO provided materials to veterinary clinics and boarding facilities to educate staff members and dog owners. An educational webinar for veterinary professionals was conducted by SAHO, WDH, and subject matter experts.

### Assessment of Changes in Canine Vaccination Practices

As part of the public health response, a survey was distributed to veterinary clinics throughout Wyoming to assess changes in canine leptospirosis vaccination rates during October 2022–January 2023 (preoutbreak) and October 2023–January 2024 (postoutbreak). Six of 10 clinics in the affected city and eight clinics in rural counties responded.

After public health outreach, 100% of clinics from the affected city that responded reported recommending the vaccine more frequently to dog owners after the outbreak (to 80.0% of owners compared with 8.3% before the outbreak). Clinic staff members also reported higher owner compliance with vaccination recommendations, estimating that the proportion of dog owners agreeing to leptospirosis vaccination increased from approximately one third (32.5%) to approximately one half (51.5%). After the outbreak, clinics reported administering leptospirosis vaccines to 33.1% of dogs seen for routine vaccination appointments, which is an increase from the previous year, when only 5.4% of dogs seen for routine vaccination appointments received leptospirosis vaccines.

Responding rural clinics reported recommending vaccination more frequently than did clinics in the affected city: approximately 50%–60% of dogs in rural clinics were reported as vaccinated both pre- and postoutbreak. These clinics also reported higher owner compliance: seven of eight rural clinics estimated that 90%–100% of clients agreed to leptospirosis vaccination for their dog when it was recommended. No leptospirosis cases were reported from rural counties during the outbreak, and just one rural clinic reported updating its vaccination protocol, changing from dog lifestyle–based recommendations to recommending vaccination of all dogs.

## Discussion

Although prevalent in temperate and tropical climates, leptospirosis has also occurred in areas with less conducive environmental conditions (*6*). Recent canine outbreaks have been reported in arid and semiarid parts of the United States, including in California and Arizona (*6*).

Canine and cattle vaccines for preventing leptospirosis are available in the United States, but human vaccines are not. Initial vaccination of dogs requires 2 doses, administered 2–4 weeks apart, with annual boosters to maintain immunity. Historically, vaccination was only recommended for dogs considered to be at increased risk for infection, based on geographic location or participation in activities that might expose them to infected animal urine. Geographically, Appalachia is considered the highest-risk region for canine leptospirosis in the contiguous United States; the upper Midwest and central Texas are also considered to be at increased risk (*8*). Lifestyle factors considered to increase dogs’ risk for exposure include contact with livestock or wildlife, time spent in kennel environments, and participation in activities that expose them to standing water or mud such as roaming farmland, hunting, hiking, or swimming (*6*,*9*). However, because of illness severity and zoonotic potential, consensus in the veterinary community is shifting to recommendation of vaccination for all dogs, and shortly after this outbreak ended, revised guidelines were published recommending that all dogs be vaccinated against leptospirosis, regardless of lifestyle or geographic location (*6*,*10*). The canine vaccine available in the United States covers four serovars: Canicola, Grippotyphosa, Icterohaemorrhagiae, and Pomona (*6*).

Health care providers should consider leptospirosis in the differential diagnosis when evaluating patients with clinically compatible illness, and inquire about occupational exposure to animals, even in historically low-risk areas. Although the patient described in this report was at increased occupational risk and demonstrated clinically compatible illness, testing was delayed because leptospirosis prevalence in the area was historically low, and the diagnosis was not considered. Early treatment can reduce disease severity and duration, and initiation of antibiotic therapy is recommended when disease is suspected, even if diagnostic test results are pending (*1*).

### Implications for Public Health Practice

A spillover event (transmission from animal to human) likely occurred in this outbreak, illustrating the importance of One Health^§§^ (i.e., a human, animal, and environmental approach) collaboration when responding to zoonotic diseases. Environmental exposure likely occurred in approximately one half of the canine cases, highlighting the need to prepare for unusually warm or wet seasons. Although many states consider human leptospirosis a reportable condition, few require notification for canine cases. The early alert by a local veterinarian of an increase in canine cases facilitated rapid investigation and interventions by public health authorities, underscoring the importance of trust between public health officials and clinical practitioners. Efforts to raise awareness of animal and human cases in this outbreak led to increased rates of canine vaccination in the affected community. Vaccination of animals can aid in controlling outbreaks and preventing spillover of leptospirosis.

## Footnotes

* The National Electronic Disease Surveillance System for Wyoming was used to monitor for additional cases.

† 45 C.F.R. part 46.102(l)(2), 21 C.F.R. part 56; 42 U.S.C. Sect. 241(d); 5 U.S.C. Sect. 552a; 44 U.S.C. Sect. 3501 et seq.

§ The patient received a diagnosis based on a qualitative immunoblot test. https://ltd.aruplab.com/Tests/Pub/0055233

¶ A probable case meets the clinical criteria and one or more supportive laboratory criteria. Clinical criteria for dogs include the onset of systemic illness within the previous 2 weeks (nonspecific fever, lethargy, polyuria, polydipsia, or clinical suspicion of acute kidney injury with or without clinical signs of leptospirosis) and two or more clinicopathologic abnormalities suggestive of a leptospirosis diagnosis. Supportive laboratory criteria for dogs include *Leptospira* microagglutination titer ≥800, detection of immunoglobulin M anti-*Leptospira* antibodies, detection of pathogenic leptospires in urine using a nucleic acid amplification test, or isolation of *Leptospira* from a clinical specimen by a reference laboratory. A confirmed case meets the clinical criteria and one or more confirmatory laboratory criteria (fourfold or higher acute to convalescent increase in *Leptospira* agglutination titer at a single laboratory, detection of pathogenic leptospires in blood using a nucleic acid amplification test, or isolation of *Leptospira* from a clinical specimen by a reference laboratory).

**
https://www.nasphv.org/Documents/VeterinaryStandardPrecautions.pdf

††
https://health.wyo.gov/rare-bacterial-infections-reported-in-wyoming/

§§
https://intranet.cdc.gov/ncezid-oho/
